# CAR‐T Cell Manufacturing for Hematological and Solid Tumors: From the Preclinical to Clinical Point of View

**DOI:** 10.1002/cam4.70726

**Published:** 2025-02-27

**Authors:** Sara Capolla, Maria Rasool, Giuseppe Toffoli, Michele Dal Bo

**Affiliations:** ^1^ Experimental and Clinical Pharmacology Unit, Centro di Riferimento Oncologico (CRO) di Aviano Istituto di Ricovero e Cura a Carattere Scientifico (IRCCS) Aviano Italy; ^2^ Doctoral School in Pharmacological Sciences University of Padua Padova Italy

**Keywords:** CAR‐T cells, FDA‐approved products, in vitro preclinical studies, manufacturing process

## Abstract

Cell therapy based on chimeric antigen receptor (CAR) T cells has represented a revolutionary new approach for treating tumors, especially hematological diseases. Complete remission rates (CRR) > 80%–97% and 50%–90% overall response rates (ORR) have been achieved with a treatment based on CAR‐T cells in patients with malignant B‐cell tumors that have relapsed or are refractory to previous treatments. Toxicity remains the major problem. Most patients treated with CAR‐T cells develop high‐grade cytokine release syndrome (CRS) and immune effector cell‐associated neurotoxicity syndrome (ICANS). However, the unprecedentedly high CRR and ORR have led to the approval of six CAR‐T cell therapeutics by the Food and Drug Administration (FDA) and the European Medicines Agency (EMA), prompting researchers to improve existing products and develop new ones. By now, around 1000 clinical trials based on CAR‐T cells are registered at ClinicalTrials.gov: 82% are for hematological diseases, while the remaining 16% are for solid tumors. As a result of this increased research, an enormous amount of conflicting information has been accumulated in the literature, and each group follows its manufacturing protocols and performs specific in vitro testing. This review aimed to combine and compare clinical and preclinical information, highlighting the most used protocols to provide a comprehensive overview of the in vitro world of CAR‐T cells, from manufacturing to their characterization. The focus is on all steps of the CAR‐T cell manufacturing process, from the collection of patient or donor blood to the enrichment of T cells, their activation with anti‐CD3/CD28 beads, interleukin‐2 (IL‐2) or IL‐7 and IL‐15 (induction of a more functional memory phenotype), and their transfection (viral or non‐viral methods). Automation is crucial for ensuring a standardized final product.

**Abbreviations**
Ab: antibody
B‐ALL: B‐cell acute lymphoblastic leukemia
B:C: beads‐to‐cell
BCMA: B‐cell maturation antigen
CAR: chimeric antigen receptor
CR: complete remission
CRISPR: cluster regulatory interspaced short palindromic repeats
CRS: cytokine release syndrome
DCs: dendritic cells
DLBCL: diffuse large B‐cell lymphoma
EGFR: epidermal growth factor receptor
ELISA: enzyme‐linked immunosorbent assay
ELIspot: enzyme‐linked immunospot
EMA: European Medicines Agency
FDA: Food and Drug Administration
FL: follicular lymphoma
GM‐CSF: granulocyte‐macrophage colony‐stimulating factor
GMP: good manufacturing practices
gRNA: guide RNA
GvDH: graft versus host disease
GZMB: granzyme B
HLA: human leukocyte antigen
ICANS: immune effector cell‐associated neurotoxicity syndrome
ICS: intracellular staining
IL: interleukin
INF‐γ: interferon‐gamma
JAK: janus kinase
mcDNA: minicircle DNA
MCL: mantle cell lymphoma
MFSA: multicolor fluorescent spot assay
MHC: major histocompatibility complex
MIP‐1: macrophage inflammatory protein
MM: multiple myeloma
NK: natural killer
NPs: nanoparticles
ORR: overall response rates
PBMCs: peripheral blood mononuclear cells
PD‐1: programmed cell‐death protein 1
RBCs: red blood cells
SB: sleeping beauty
scFv: single‐chain variable fragment
SMASH: small molecule‐assisted shutoff
SOC: standard of care
STAT3: signal transducer and an activator of transcription 3
SWIFF: switch‐off
TAA: tumor‐associated antigens
TCM: central memory T cells
TCR: T‐cell receptor
TME: tumor microenvironment
TNFR: tumor necrosis factor receptor
TNF‐α: tumor necrosis alpha
TRAIL: TNF‐related apoptosis‐inducing ligand
TRUCK: T cells redirected for universal cytokine killing
TSA: tumor‐specific antigens
TSCM: memory T stem cell
VH: variable heavy chain
VL: variable light chains

## 1 Background

In recent decades, cell therapy based on chimeric antigen receptor (CAR) T cells has revolutionized the treatment of cancers, particularly hematological diseases. CAR‐T cells are lymphocytes T that have been engineered to recognize a specific antigen and are made up of four components. The affinity and specificity of CAR are determined by the first external portion, which is responsible for binding to the target antigen. It typically consists of the single‐chain variable fragment (scFv) domain, which is formed by the in‐frame fusion of the variable heavy (VH) and light (VL) chains of an antibody (Ab) with a linker. Recently, a single domain (typically VL) has been proposed as a promising and more efficient external portion for CAR constructs compared to scFvs [1]. The second component is the hinge region, which consists of the IgG1 or IgG4 hinge‐CH2‐CH3 Fc domains or portions of CD4, CD8, CD3ζ, or CD28 [2] and is fundamental for the activation of CAR‐T cells against the target cancer cell [3]. The hinge region also connects scFv to the transmembrane region, the third component of the CAR construct. The latter regulates CAR signaling and controls CAR expression on the T cell surface [4]. The final component of the CAR construct is the intracellular signaling domain, whose composition varies from one generation of CAR‐T cells to another. In all instances, the CD3ζ signaling domain is present.

The strength of CAR technology is the combination of specificity for the target antigens and strong cytotoxicity against tumor cells. Over generations, the intracellular domain has been modified to overcome toxicity and improve efficiency. The first generation is the simplest; it comprises the extracellular and transmembrane domains and only CD3ζ as the intracellular signaling domain.

A costimulatory domain (like CD28 or 4‐1BB) is added in the second generation to boost T‐cell proliferation and expansion after repeated exposure of T cells to the antigen. In the third generation, both CD28 and 4‐1BB costimulatory domains are present. The fourth generation of CAR‐T cells is known as “T cells redirected for universal cytokine killing (TRUCK)” and is characterized by the addition of a constitutive or inducible expression cassette for cytokine production (e.g., interleukin‐12 (IL‐12)) released by CAR‐T cells [5] to modulate the T‐cell response, improve the activation of natural killer (NK) cells, and polarize macrophages to the M1 phenotype against the tumor cells [6]. IL‐12 is not the only cytokine used in this context; IL‐2, IL‐7, IL‐10, IL‐15, IL‐18, IL‐23, and IL‐27 have also been evaluated [7]. Constructs in fifth‐generation CAR‐T cells have a truncated version of the cytoplasmic IL‐2 receptor β‐chain domain that can bind to the transcription factor signal transducer and activator of transcription 3 (STAT3). The activation of T cells occurs when they bind to the antigen, which simultaneously triggers TCR (via CD3ζ domain), costimulatory domains (CD28 domain), and cytokine Janus kinase (JAK)‐STAT3/5 signaling [5]. The IL‐2 receptor β‐chain domain and the JAK–STAT pathway promote cell proliferation and prevent terminal differentiation, leading to better persistence and higher therapeutic effects in leukemia [8].

CAR‐T cell therapies have resulted in unprecedentedly high complete remission (CR) rates, particularly in patients with B‐cell acute lymphoblastic leukemia (B‐ALL) and diffuse large B‐cell lymphoma (DLBCL). Compared to adoptive T‐cell therapies like T‐cell receptor (TCR) engineering, CAR‐T cells offer several advantages. First, CAR‐T cells are not restricted to human leukocyte antigen (HLA) for antigen presentation, increasing the number of patients who can receive treatment. Second, CAR‐T cells receive a strong signal even when the antigen concentration is low, resulting in the rapid proliferation of T cells and higher tumor killing than TCR T cells [9]. The success of CAR‐T cells prompted the Food and Drug Administration (FDA) [10] to approve six CAR‐T cell‐based therapies.

Kimriah (tisagenlecleucel, tisa‐cel) was the first cell‐based therapy approved by the FDA and European Medicines Agency (EMA) for the treatment of relapsed/refractory (r/r) pediatric and young adult ALL (FDA approval in 2017, EMA approval in 2018), r/r DLBCL (approved in 2018), and r/r follicular lymphoma (FL, approved in 2022) with reported overall response rates (ORR) of 82%, 53%, and 83%, respectively (Table 1) [11]. The target of tisa‐cel is CD19, an appealing antigen uniformly expressed by most B‐cell malignancies, present only on the surface of healthy precursor cells, mature B cells, and plasma cells [12]. The impressive ORR was matched by a significantly higher CR rate for FL‐affected patients after ≥ 2 lines of systemic therapy, as shown in the ELARA cohort (CR tisa‐cel vs. standard of care (SOC): 69% vs. 18%) [13]. Patients with DLBCL are not subjected to this. Although tisa‐cel has a higher ORR than SOC, the CR is the same (second‐line therapy: tisa‐cel vs. SOC: 28% vs. 28%—BELINDA study) [14].

**TABLE 1:** Details of FDA/EMA‐approved CAR‐T cells.

CD19‐CAR‐T cells				
Commercial and product name	Company	FDA(EMA) approval	Indication	Efficacy
Kymriah Tisagenlecleucel (Tisa‐cel)	Novartis	2017 (2018)	r/r Pediatric and young adult ALL	ORR: 82%
		2018 (2018)	r/r DLBCL	ORR: 53% CR tisa‐cel vs. SOC: 28% vs. 28% (BELINDA study)
		2022 (2022)	r/r FL	ORR: 86% CR tisa‐cel vs. SOC: 69% vs. 18% (ELARA cohort)
Yescarta Axicabtagene ciloleucel (Axi‐cel)	KITE Pharma	2017 (2018)	r/r DLBCL—transplant candidates	ORR: 70% CR axi‐cel vs. SOC: 65% vs. 32% (ZUMA‐7 study)
Tecartus Brexucabtagene autoleucal (Brexu‐cel)	KITE Pharma	2020 (2020)	r/r MCL	ORR: 85%
Breyanzi Lisocabtagene maraleucel (Liso‐cel)	JUNO Therapeutics	2021 (2022)	r/r DLBCL after ≥ 2 systemic treatments	ORR: 79% CR liso‐cel vs. SOC: 66% vs. 39% (TRANSFORM study)
		2021 (2022)	FL	ORR: 97% CR: 94% (TRANSCEND FL study)
**BCMA‐CAR‐T cells**				
**Commercial and product name**	**Company**	**Approval FDA (EMA)**	**Indication**	**Efficacy**
Abecma Idecabtagene vicleucel (Ide‐cel)	Celgene Corp.	2021 (2021)	MM	ORR: 71% CR ide‐cel vs. SOC: 39% vs. 5% (Randomized open‐label study)
Carvykti Ciltacabtagene autoleucel (Cilta‐cel)	Janssen Biotech	2022	MM	ORR: 84.6% CR cilta‐cel vs. SOC: 73% vs. 22% (Randomized open‐label study)

*Note:* CD19‐ and BCMA‐CAR‐T cell products were divided into groups. In the table, the commercial and product names, the producing company, the year of FDA and EMA approval, the therapeutic indication, and the efficacy are reported.

Abbreviations: ALL, acute lymphoblastic leukemia; CR, complete response; DLBCL, diffuse large B‐cell lymphoma; FL, follicular lymphoma; MCL, mantle cell lymphoma; MM, multiple myeloma; ORR, overall response rate; r/r, relapsed/refractory; SOC, standard of care.

The FDA and EMA have approved additional CD19‐CAR‐T cell products like Yescarta (axicabtagene ciloleucel; axi‐cel) and Breyanzi (lisocabtagene maraleucel, liso‐cel) as second‐line treatments of DLBCL due to the potential of this approach [15]. The ORR rates of both cell therapies are promising: axi‐cel ORR is 70% and liso‐cel ORR is 79%. Notably, the CR rates induced by axi‐cel and liso‐cel were significantly higher than SOC: 65% for axi‐cel compared to 32% for SOC in the ZUMA‐7 study [16]; and 66% for liso‐cel compared to 39% for SOC in the TRANSFORM study [15, 16]. The FDA and EMA have approved the same approach for treating different CD19+ diseases, like mantle cell lymphoma (MCL). The CD19‐CAR‐T product called Tecartus (brexucabtagene autoleucal, brexu‐cel) resulted in an ORR of 85%, with 59% of patients achieving CR [17].

Another popular target for CAR‐T cell‐based therapy is the B‐cell maturation antigen (BCMA), a member of the tumor necrosis factor receptor (TNFR) superfamily, which is preferentially expressed on healthy mature B lymphocytes. Overexpression of BCMA has been linked to the progression of multiple myeloma (MM) [18]. Two cell therapies have been developed to treat MM‐affected patients after four or more prior lines of therapy: Abecma (idecabtagene vicleucel—ide‐cel) and Carvykti (ciltacabtagene autoleucel—cilta‐cel). Both CAR‐T cell products are effective against MM, leading to an ORR of over 70% and a significant increase in CR rates compared to SOC (CR ide‐cel vs. SOC: 39% vs. 5% [19]; CR ide‐cel vs. SOC: 73% vs. 22% [20]).

The success of CAR‐T cells has led to the registration of approximately 1000 clinical trials at ClinicalTrials.gov. Most of these trials are now in Phase 1, while only six are in Phase 4. Among the newly introduced therapies, 82% targeted hematological diseases, while the other 16% were aimed at solid tumors [21]. The focus on liquid tumors primarily arises from challenges in identifying targetable antigens, tumor heterogeneity, tumor infiltration, and the immunosuppressive nature of the tumor microenvironment (TME) commonly found in solid tumors. The heterogeneity of solid tumors presents a challenge in selecting effective targeting molecules; primary and metastatic cancer cells can exhibit different expression profiles, and this variation can also occur among patients diagnosed with the same cancer [22]. Moreover, the unfavorable TME, which is characterized by hypoxia and elevated concentrations of immunosuppressive soluble factors, as well as various cells (e.g., myeloid cells and regulatory T cells) and proteins (e.g., programmed cell death protein 1 (PD‐1)) [23], hinders T cell activation [24] and prevents the infiltration of CAR‐T cells into the tumor site.

The efficacy of CAR‐T cell therapy is indisputable; however, this treatment approach has its limitations. Only a limited number of patients who received the therapy achieved CR. Concurrently, numerous patients suffered from severe toxicities, including cytokine release syndrome (CRS), immune effector cell‐associated neurotoxicity syndrome (ICANS), and, in some instances, death. In addition, a significant number of patients (up to 66%) experience relapse following treatment [25].

The success of CAR‐T cell therapy is heavily influenced by the manufacturing process. Every step poses a challenge and can be further improved: the collection of autologous or allogeneic T cells, along with their pre‐infusion exhausted state, strongly impacts the efficacy of CAR‐T cell therapies [26]. Overstimulation of T cells in vitro before infusion triggers their early exhaustion [27], and the method of transfection can result in the malignant transformation of T lymphocytes. Therefore, due to the large amount of conflicting published information regarding the manufacturing methods for CAR‐T cell production and subsequent in vitro characterization, this review provides a summary of in vitro CAR‐T cell purification, expansion, and characterization methods, focusing particularly on FDA‐approved protocols.

## 2 Selection of the Starting Material

### 2.1 Autologous and Allogeneic Transplantation

The initial step of CAR‐T cell manufacturing involves selecting and collecting the starting material. In autologous CAR‐T cell therapy, T cells are extracted from a patient and re‐infused into the same individual after being engineered in the lab; on the other hand, in allogeneic transplantation, commonly referred to as “off‐the‐shelf/healthy donor,” T lymphocytes are sourced from a healthy donor and administered to a cancer patient. Both procedures come with their own advantages and disadvantages. With autologous transplantation, there is no risk of graft versus host disease (GvHD), and the persistence of cells in vivo tends to be longer than that observed with allogeneic transplantation [28]. However, autologous (patient‐derived) T‐cell therapy poses several challenges, including a complex manufacturing process, high cost, T‐cell variability, T‐cell exhaustion, and dysfunction due to previous tumor therapies. The efficacy of CAR‐T cells heavily depends on the characteristics of the purified T lymphocytes, which can be influenced by factors such as the patient's age, the type of cancer, prior treatments received, and the risk of contamination of T cells with cancer cells. To address some of the challenges associated with autologous CAR‐T cell transplantation, allogeneic CAR‐T cell therapy has been proposed as a potential solution. Allogeneic therapies offer a homogeneous, standardized, and readily available option at a lower cost, with the potential of multiple dosing. Most notably, allogeneic CAR‐T cell therapy may be especially beneficial for aggressive tumors, as it eliminates the risk of product contamination with malignant T cells [29, 30]. However, limitations of allogeneic transplantation include the risk of GvHD and host‐mediated immune rejection, during which the recipient's immune cells attack the infused CAR‐T cells, diminishing their efficacy [31]. Numerous strategies have been developed to mitigate these issues. For instance, host CD52+ alloreactive T cells, which are responsible for depleting injected CAR‐T cells, can be eliminated using anti‐CD52 antibodies, such as alemtuzumab. To implement this, CAR‐T cells must be genetically edited to disrupt the endogenous expression of CD52 before infusion. Genetic editing can also target the TCR and HLA to further reduce alloreactivity [32]. Another proposed strategy is to harvest different subsets of T lymphocytes, such as those carrying a γδ TCR, due to their increased cytotoxicity compared to the conventional αβ TCR used in CAR‐T cell products [28]. Finally, reinfusing less differentiated populations, such as pluripotent stem cells, progenitor T cells, or cord blood cells, may result in reduced GvHD [31].

### 2.2 T‐Cell Enrichment Methods

The starting material for CAR‐T cell therapy is the peripheral blood mononuclear cells (PBMCs) from the patient or donor. PBMCs consist of 70%–90% lymphocytes (which include 70%–85% CD3+ T cells, 5%–10% B cells, and 5%–20% NK), 10%–20% monocytes, and 1%–2% dendritic cells (DCs) [33]. PBMCs are separated from whole blood through leukapheresis or density gradient centrifugation (Ficoll‐Paque) and then washed to remove contaminants such as anticoagulants, red blood cells (RBCs), and platelets. Removing platelets is crucial to avoid false results in flow cytometry, as platelets can aggregate with immune cells and express Fc receptors that may be labeled by fluorescent Abs [34].

**FIGURE 1:**
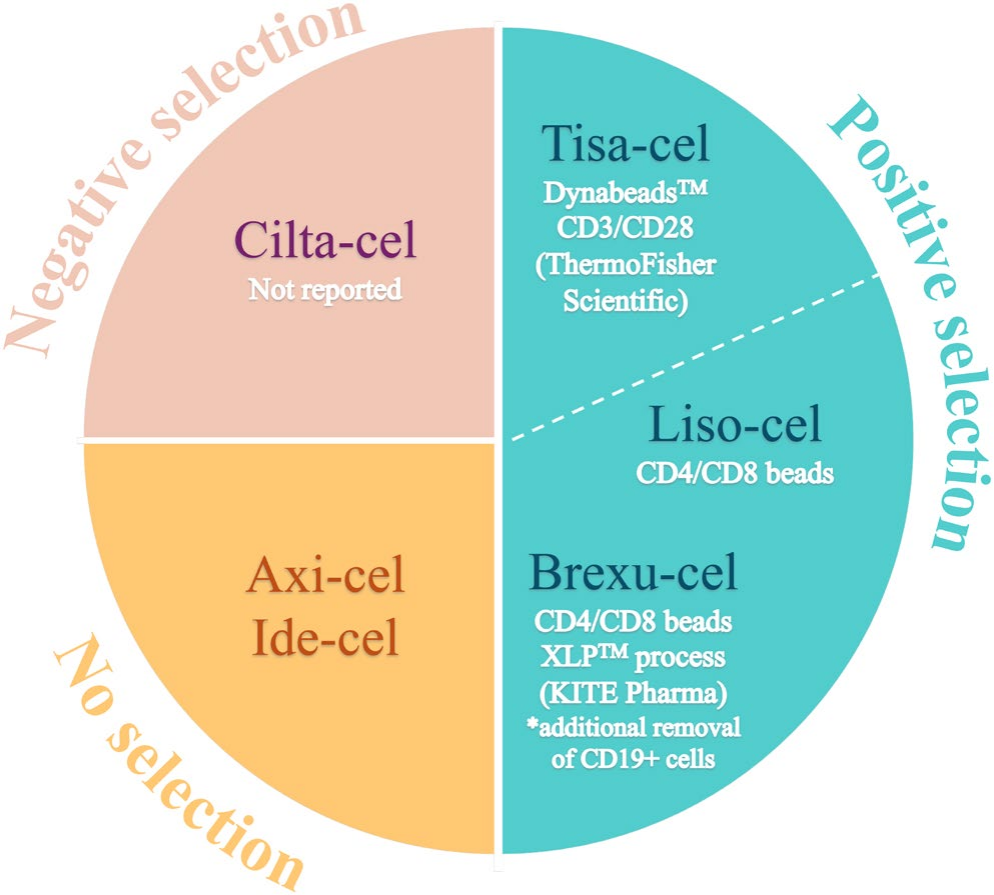
Schematic representation of the strategies used to select T cells in clinically approved products.

The second step in CAR‐T cell production involves enriching the T‐cell population through selection, although this step is optional. Positive or negative selection methods utilize paramagnetic beads labeled with Abs. Anti‐CD4/CD8 [35, 36, 37, 38, 39, 40] or anti‐CD3 Abs‐conjugated beads have been employed in these processes [1, 41]. The validity of these procedures is supported by the inclusion of positive selection in three out of six FDA‐approved CAR‐T cell products. Specifically, brexu‐cel [42] and liso‐cel [43] result from positive selection using anti‐CD4/CD8 Ab‐conjugated beads, whereas tisa‐cel [44] comes from positive selection with anti‐CD3/CD28 Ab‐conjugated beads (Figure 1).

Positive selection allows for the choice of Abs used to enrich the T lymphocyte population. However, whenever a receptor is bound, the cell can receive a signal, which may alter its behavior. Shah et al. [18] have shown that CD8 and CD4 binding can affect T‐cell biology by increasing the release of inflammatory cytokines and enhancing toxicity compared to CD3/CD28 enrichment. In addition, CD3/CD28 selection also enables the purification of γδ T cells, which are excluded by CD4/CD8 selection due to the absence of these markers on their surface. The importance of γδ T cells lies in their strong association with favorable prognosis and their natural anti‐tumor function [40].

Negative selection presents a valid alternative for enriching T lymphocytes, resulting in CAR‐T end products with similar expansion, transduction efficiency, and phenotype when compared to those obtained through CD4/CD8 positive selection [40]. This process uses a cocktail of anti‐CD14, anti‐CD15, anti‐CD16, anti‐CD19, and anti‐CD56 Ab‐conjugated beads to remove monocytes/macrophages, granulocytes, NK cells, and B cells from PBMCs, leaving T lymphocytes untouched. Negative selection has been used in clinical (cilta‐cel) (Figure 1) [45] and preclinical contexts (Table 2) [40, 46, 47, 48, 49, 50].

**TABLE 2:** T cell selection methods for CAR‐T cell therapy in preclinical studies.

Positive selection of CD4+ and CD8+ cells				
Reagents used	Target antigen and disease	CD4/CD8 ratio	Preclinical in vivo efficacy	References
CliniMACS CD4 Reagent System and 2‐step CliniMACS procedure (Miltenyi Biotec)	CD19—ALL *Additional negative selection of CD8+ TCM cells	1	Remission rate 93% (patients)	[35]
CliniMACS procedure (Miltenyi Biotec)	CD19—ALL	Not reported	100% CR	[39]
CD4 and CD8 Isolation Kits (Miltenyi)		1:1	40% survival	[37]
CilinMACS CD4 and CD8 reagents (Miltenyi)	CD19—NHL	1–2.5	Significant decrease of tumor mass	[38]
GMP‐grade CliniMACS CD4 and CD8 reagents (Miltenyi Biotec)	CD20—transfected HEK293T cells	3.3–4.2	40% cytotoxicity in vitro	[36]
CliniMACS Plus (Miltenyi Biotec)	CD22—ALL	2–6	Increased survival	[40]
**Positive selection of CD3+ cells**				
**Reagents used**	**CAR‐T cells developed**	**CD4/CD8 ratio**	**Efficacy**	**References**
Mojosort Human CD3 T‐cell isolation kit (BioLegend)	HER‐2—breast cancer	Not reported	30%–80% survival	[1]
Microbeads (Miltenyi Biotec)	CD26—T‐cell lymphoma *Additional positive selection of CD34+ cells	Not reported	Decrease of xenograft tumor progression 50% CR	[41]
**Negative selection**				
**Reagents used**	**CAR‐T cells developed**	**CD4/CD8 ratio**	**Efficacy**	**References**
Pan T Cell Isolation Kit (Miltenyi Biotec)	PDL‐1—NSCLC, gastric cancer, HCC (patient‐derived cells)	Not reported	Decrease of tumor mass growth	[46]
Pan T Cell Isolation Kit II (Miltenyi Biotec)	CD19—Burkitt's Lymphoma	Not reported	100% CR	[47]
EasySep Human T‐cell enrichment kit (Stemcell Technologies)		Not reported	Significant decrease of tumor burden	[48]
EasySep Human T Cell Isolation kit (Stemcell)	CD20—Burkitt's Lymphoma	Not reported	Increased survival	[49]
RoboSep‐C platform	CD22—ALL	2:1–6:1	Increased survival	[40]
CD4 + CD8+ negative isolation kit (STEMCELL Technologies)	GPC3 – HCC	Not reported	100% CR	[50]
**Absence of selection**				
**CAR‐T cells developed**		**CD4/CD8 ratio**	**Efficacy**	**References**
5 T4—Ovarian cancer		Not reported	75%–80% CR	[51]
CD19—Hela‐transfected cells		5:1	Cytotoxicity in vitro: 40%	[23]
CD19 and gp350—BL		1:4 and 1:2	CD19.CAR‐T: significant delay in tumor distribution	[52]
CD19—ALL		2.5	Increased OR	[53]
		Not reported	80% tumor‐free survival mice	[54]
CD19—Burkitt's lymphoma		Not reported	Improved OS	[55]
CD20—Lymphoma		Healthy donors: 4 MCL‐affected patients: 2	Efficient eradication of tumors	[56]
CD38—MM		Not reported	Decreased tumor growth	[57]
CD138—MM		Not reported	Higher OS	[58]
NKGD2—Colorectal cancer		Not reported	Increased survival	[59]
PSCA—Prostate cancer		Not reported	Significant decreased tumor growth	[60]
HER‐2—Breast cancer		Not reported	Controlled development or disappearance of tumor mass	[61]
HER‐2—Breast cancer		Not reported	Increased survival	[62]
HER‐2—Breast cancer		Not reported	High in vitro cytotoxicity	[63]

*Note:* When not otherwise stated, efficacy refers to in vivo studies made in tumor‐bearing mice.

Abbreviations: ALL, acute lymphoblastic leukemia; CAR, chimeric antigen receptor; CR, complete response; GMP, good manufacturing practice; HCC, hepatocellular carcinoma; HER‐2, human epidermal growth factor receptor 2; MACS, magnetic cell separation; MCL, mantle cell lymphoma; MM, multiple myeloma; NHL, non‐Hodgkin lymphoma; NKGD2, natural killer group 2 member D; NSCLC, non‐small cell lung cancer; OS, overall survival; PDL‐1, programmed cell death ligand 1; TCM, T central memory.

As T cells make up the majority of PBMCs, many research groups opt to stimulate them directly without specifically enriching the T cell population [23, 51, 52, 53, 54, 55, 56, 57, 58, 59, 60, 61, 62, 63]. This method's feasibility has also been supported by the FDA's approval of CAR‐T cell products like axi‐cel [64] and ide‐cel (Bristol‐Myers Squibb Prescribing Information), which are developed from the direct stimulation and transduction of PBMCs. When no selection procedure is applied, it is important to consider the dose‐dependent effects that monocytes have on the expansion and transduction of both normal T lymphocytes [65] and CAR‐T cells [66].

The literature extensively documents both positive and negative selection, as well as the direct use of PBMCs; yet, there are no reported differences in efficacy between these approaches (Table 2).

## 3 T‐Cell Activation and Differentiation In Vitro

### 3.1 Activation of T Cells With Antibodies or Antibody‐Conjugated Beads

The production of CAR‐T cells typically involves a three‐step process: activation, followed by transduction and expansion ex vivo for at least 6 days. In their natural state, CD8+ and CD4+ T cells become activated, proliferate, and differentiate into various short‐lived effector subsets after interacting with a target antigen that is bound to major histocompatibility complex (MHC) class I or MHC class II molecules. In vitro, this activation step can be achieved using free or bead‐coated anti‐CD3/CD28 Abs. The latter approach is the most common method for in vitro T cell activation, accounting for 66% of CAR‐T cell products [67]. Among the six FDA‐approved CAR‐T cell products, three utilized anti‐CD3/CD28 Abs for T‐cell activation: brexu‐cel [42], axi‐cel [68], and ide‐cel (www.fda.gov/media/147055/download). The use of bead‐coated anti‐CD3/CD28 Abs is the predominant method employed in preclinical studies for T‐cell activation. Research has explored various beads‐to‐cell (B:C) ratios to optimize this process. A B:C ratio of 3:1 is the most commonly reported condition [39, 40, 69, 70, 71, 72]; however, other B:C ratios, such as < 1:1 [73], 1:1 [48, 50, 51, 74, 75], 2:1 [50], 3:1 [40], and 5:1 [76, 77, 78], have been noted as effective.

When T lymphocytes are activated by binding to their corresponding antigen, they upregulate and express surface markers such as CD25 and CD69. CD25 is recognized as a universal marker for late activation; it is part of the IL‐2 receptor and is highly expressed on activated lymphocytes and regulatory T cells [69]. The expression of CD25 correlates with the magnitude of activation [77]. In contrast, CD69 is rapidly induced following TCR/CD3 engagement and plays a crucial role in determining patterns of cytokine release, homing, and migration. CD69 is considered an early activation marker, detectable 30–60 min after activation, and its levels decrease after 4–6 h [70]. Despite the significance of these markers, only a few authors have reported the percentage of activated cells after exposure to beads, and the results have been inconsistent. Reported percentages for CD3 + CD25+ range from 10% to 90% [40, 75, 76], while CD3+ CD69+ cells [75, 76] range from 30% to 80%.

### 3.2 Differentiation of T Cells In Vitro

Cytokines play a central role in regulating the immune response, including the fight against cancer. Tumor cells secrete cytokines that promote their proliferation while inactivating anti‐tumor T cells infiltrating the TME. The same cytokines attract immune cells to invade the TME and trigger a pro‐inflammatory state, which serves as the first line of defense against the tumor. In the next phase, chronic inflammation transforms the TME from being hostile to tumors into one that promotes tumor growth. The balance between pro‐inflammatory and anti‐inflammatory cytokines governs these processes [71]. Additionally, cytokines are vital for the quality and functionality of CAR‐T cells. IL‐2, IL‐7, and IL‐15 influence the composition, quality, and phenotype of transferred T cells, significantly affecting the in vivo efficacy of CAR‐T cell‐based therapies [71]. They also induce T‐cell proliferation in vitro [79, 80].

IL‐2 has been widely used in the manufacturing protocols for CAR‐T cell production, including for FDA‐approved therapies such as brexu‐cel [42] and axi‐cel (300 U/mL) [68]. Various IL‐2 concentrations have been tested in the published studies, with 300 [46, 81, 82, 83] and 100 U/mL [1, 51, 74, 78, 81] being the most commonly used for T cell activation, compared to lower concentrations like 20 [76], 40 [40], 50 [35, 53, 78], and 200 U/mL [41, 49].

Despite its widespread use, IL‐2 can lead a relatively mature phenotype (CD62L^low^CCR7^+^CD27^+^CD28^+^) that favors the expansion of regulatory T cells rather than memory T cells. Therefore, it is crucial to select a population enriched with memory cells, particularly memory T stem cells (TSCM). These cells are important because they demonstare better persistence, engraftment, and efficacy in vivo compared to more differentiated phenotypes, such as effector T lymphocytes [37, 84]. The enhanced efficacy of TSCM is largely due to their ability to undergo numerous cell divisions, similar to stem cells, which remain in a quiescent state but can rapidly generate highly proliferative, self‐renewable, multipotent progeny that respond rapidly to their corresponding antigens. The proliferation and survival of T cells correlate with anti‐tumor efficacy and persistence in vivo, making TSCM a promising focus for CAR‐T cell‐based therapies [72]. Consequently, CAR‐T cell production methods have evolved to enrich the TSCM and central memory T‐cell (TCM) populations. This is achieved by limiting T‐cell proliferation in vitro before transplantation and by stimulating receptors for IL‐7 and IL‐15, which can mitigate the terminal differentiation of T cells and increase the proportion of memory stem cells. The addition of IL‐7 and IL‐15 to the culture media results in lower expression of exhaustion markers, a higher secretion of pro‐inflammatory cytokines, and a stronger anti‐tumor effect of the stimulated T cells compared to those stimulated with IL‐2 [71]. However, despite these encouraging results, the optimal cytokine composition for promoting TSCM enrichment has not been clearly defined. There is currently no consensus in the literature regarding the use of IL‐7 and IL‐15; some authors even report that IL‐2 stimulation can induce a TSCM phenotype [72, 81]. Moreover, the proportion of TSCM achieved through stimulation with IL‐7 and IL‐15 can vary significantly, ranging from 10%–30% to 60%–70% [37, 38, 39, 85], potentially affecting the efficacy of CAR‐T cells.

The search for consensus is further complicated by the fact that the percentage of TSCM achieved after stimulation is often not reported in most published studies [24, 63, 72, 74, 83].

## 4 Transduction of Activated T Cells

To produce CAR‐T cells, T lymphocytes are genetically modified to express the CAR. To date, viral and non‐viral methods for gene transfer have been developed.

### 4.1 Viral Transduction Methods

**FIGURE 2:**
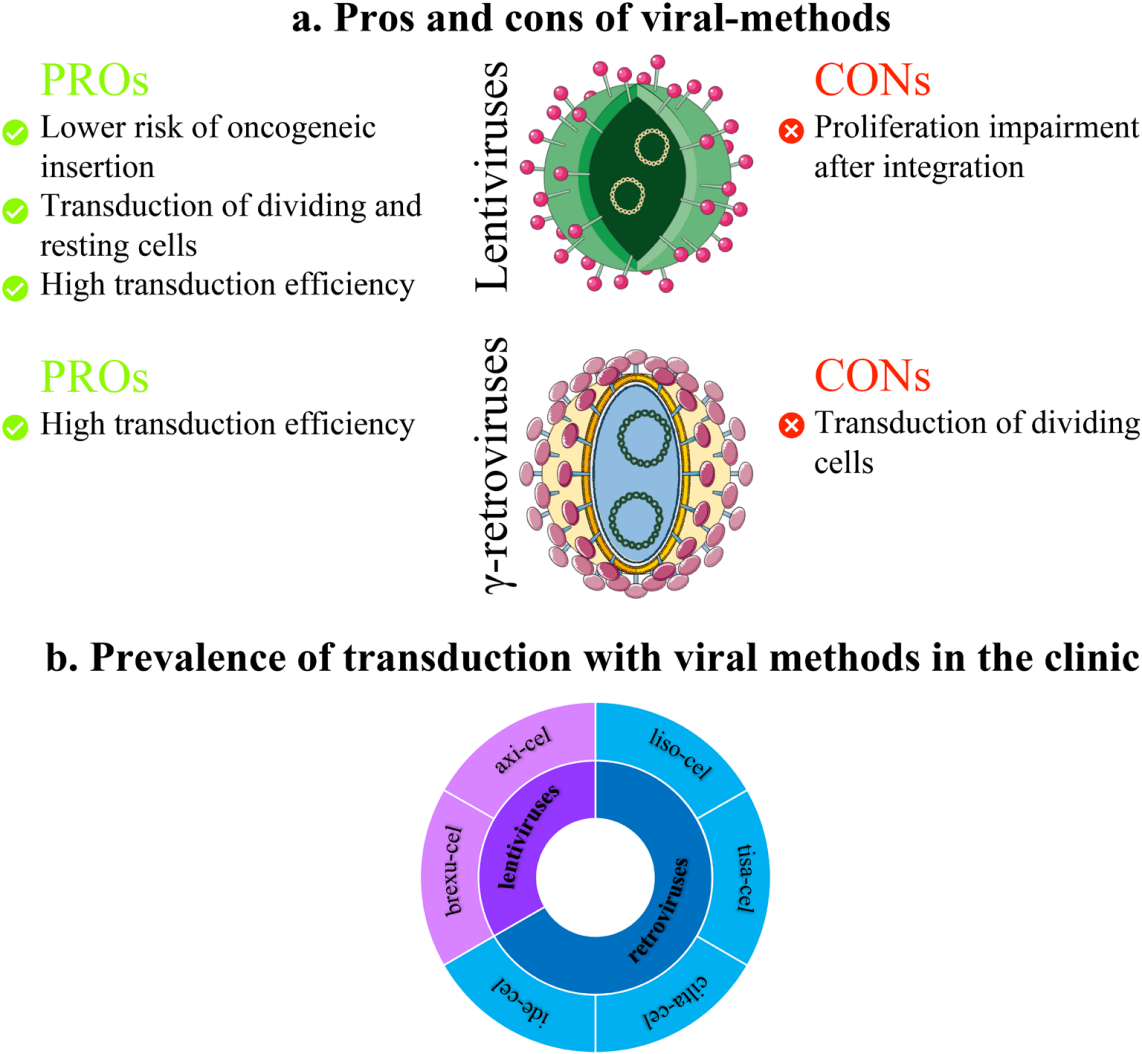
Schematic representation of viral methods for CAR‐T cell manufacturing. (a) List of pros and cons of the CAR‐T cell transfection with viral methods. (b) Prevalence of transduction with viral methods in the clinical context. The figure was partly generated using Servier Medical Art, provided by Servier, licensed under a Creative Commons Attribution 3.0 unported license. CAR, chimeric antigen receptor; CRISPR, regulatory interspaced short palindromic repeats.

Viruses have become the standard clinical method for the genetic modification of CAR‐T cells. This method ensures the integration of genetic material into the host cell genome, achieving high transduction rates and long‐term stable expression of the transgene. The two main classes of viral vectors are γ‐retroviruses and lentiviruses. γ‐retroviruses only transduce dividing cells, making activation necessary, while lentiviruses can transduce both dividing and resting cells (Figure 2a) [67, 86]. Among the six CAR‐T cell products approved by the FDA, 33% (axi‐cel and brexu‐cel) were derived using γ‐retroviruses, whereas 67% were based on lentivirus‐mediated transduction [87] (Figure 2b). In preclinical studies, strategies using both lentiviruses [24, 37, 39, 40, 41, 46, 47, 49, 50] and retroviruses have been widely reported [58, 63, 74, 75, 88, 89, 90].

Currently, there is no substantial evidence indicating that one viral vector is significantly better than the other. A major concern with viral vectors is the potential risk of oncogenic gene insertion (https://www.fda.gov/vaccines‐blood‐biologics/safety‐availability‐biologics/fda‐investigating‐serious‐risk‐t‐cell‐malignancy‐following‐bcma‐directed‐or‐cd19‐directed‐autologous). However, no cases have been documented in clinical settings. Additionally, the typical production of lentiviruses through transient transfection, which requires large amounts of DNA, can increase production costs [67]. Lentiviruses tend to integrate into the introns of transcriptionally active genes, which reduces the risk of oncogenic insertion. In contrast, γ‐retroviruses preferentially integrate near transcription start sites, enhancers, and promoters. The use of lentiviruses is not without risk, particularly because their integration into the T‐cell genome can impact T‐cell proliferation and influence the overall success of CAR‐T cell therapies. Indeed, when integration occurs within transcription units, it can hinder cell growth. In contrast, integration outside these transcription sites is often associated with more effective therapies. However, in this latter scenario, it is important to consider the effects of pro‐proliferative modifications and their implications for patient safety [91].

### 4.2 Non‐Viral Methods to Generate CAR‐T Cells

Stable gene transfer delivery can be achieved through various non‐viral methods, including transposons, clustered regulatory interspaced short palindromic repeats (CRISPR)/Cas9, minicircle DNA (mcDNA) delivery vectors, and nanoparticles (NPs).

**FIGURE 3:**
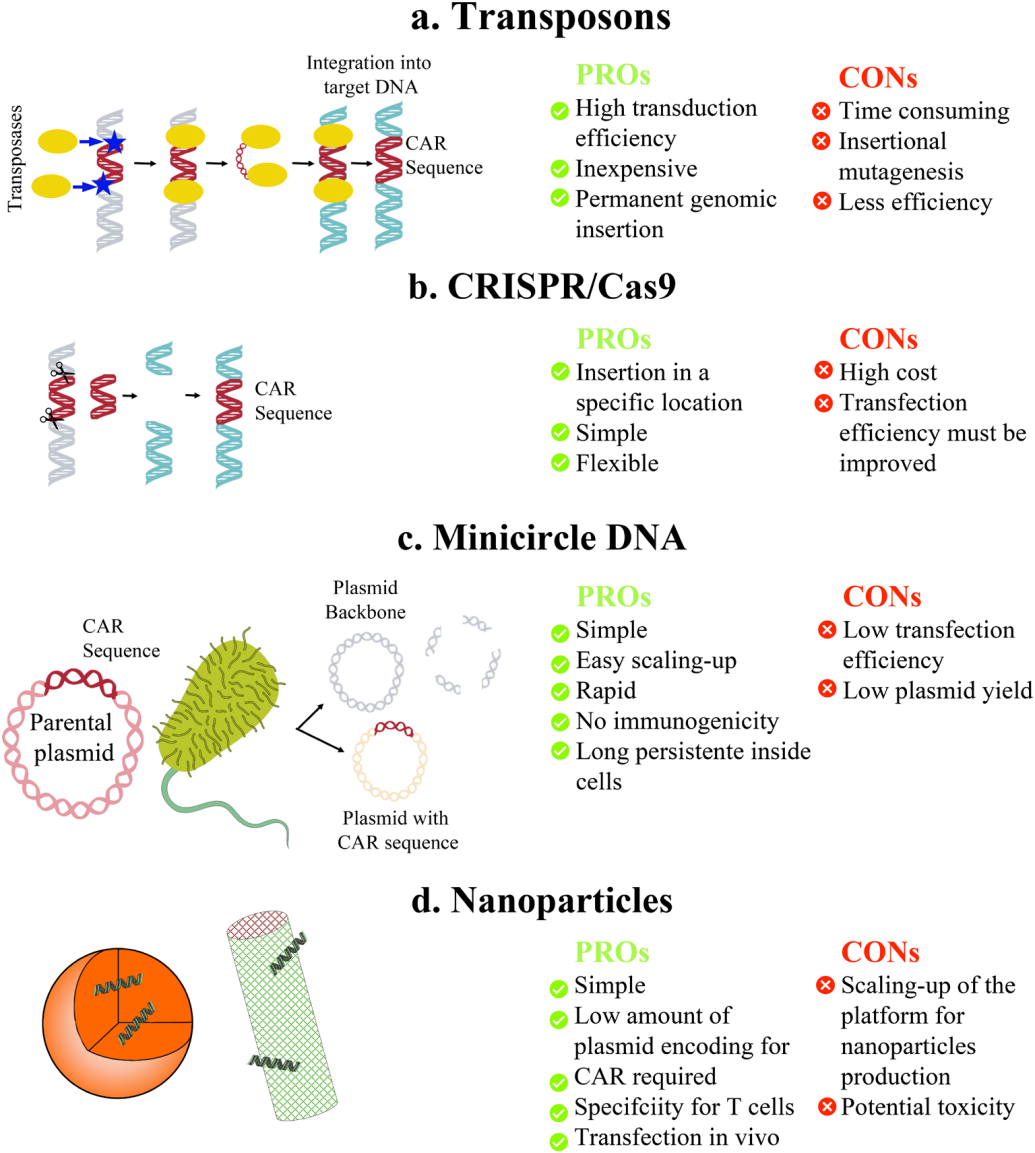
Pros and cons of non‐viral methods for CAR‐T cell manufacturing. List of pros and cons of non‐viral methods for the transfection of CAR constructs: (a) Transposons, (b) CRISPR/Cas9, (c) minicircle DNA, (d) nanoparticles. The Figure was partly generated using Servier Medical Art, provided by Servier, licensed under a Creative Commons Attribution 3.0 unported license. CAR, chimeric antigen receptor; CRISPR, regulatory interspaced short palindromic repeats.

Transposons are DNA sequences capable of changing their position within a genome through a cut‐and‐paste mechanism. This process relies on transposons and transposases. A transposon carries the target gene flanked by inverted terminal repeats, which are bound by transposase. The enzyme mobilizes the entire transposon in the target genome. Advantages of transposons include low production costs, the absence of a need for clinical grade vectors, efficient and permanent genomic insertion, and higher biosafety compared to viral methods, as they do not integrate in a biased manner. In addition, transposon transfection can be performed in both activated and resting primary T cells [67, 87]. In the latest generations of CAR‐T cells, simultaneous transfer of multiple genes is crucial. Transposons are particularly suitable for this purpose because they can carry larger genetic cargoes than viruses [92]. As a result, transposons have been extensively studied, yielding promising preclinical and early clinical outcomes [67, 87, 93]. However, transposons have some disadvantages, such as the longer time required to generate CAR‐T cells compared to viral methods and lower transfection efficiencies. The two most commonly used transposon/transposase systems—Sleeping Beauty (SB) and piggyBac—enable transfection efficiencies of 25%–80% [61, 94, 95, 96, 97] and 20%–40% of T cells [53, 61, 98], respectively. Additionally, insertional mutagenesis and subsequent malignant transformation have been observed in CAR‐T cells produced using piggyBac (Figure 3a) [99].

CRISPR/Cas9 technology represents a promising alternative to transposons, achieving CAR+ cell percentages of around 20%–60% [52, 100, 101, 102]. This technology, derived from the immune system of bacteria and archaea, utilizes short RNA fragments (guide RNA or gRNA) that are complementary to target DNA sequences, guiding the Cas9 enzyme to those specific sequences. Cas9 then cuts the original DNA, facilitating the introduction of new genes using the cell's DNA repair machinery [103]. CRISPR/Cas9 is a simple, flexible, and precise method for integrating multiple transgenes at a predefined location within the genome (Figure 3b). Current challenges include optimizing and standardizing the gene editing process to improve stability and efficiency, as well as reducing or eliminating chromosome loss, a recently discovered consequence of genome editing [104].

Another effective non‐viral mechanism for transfecting CAR constructs into T cells involves using CAR mcDNA vectors combined with electroporation. These mcDNA vectors originate from a supercoiled parental DNA vector containing the CAR sequence. After transformation into ZYCY10P3S2T E. coli, the parental vector undergoes recombination, resulting in two products: the CAR mcDNA vector and a plasmid backbone containing bacterial origin and antibiotic genes. The latter is degraded by I‐SceI endonuclease following the addition of L‐arabinose to the culture medium. The advantages of mcDNA vectors include a lack of immune response due to the absence of residual bacterial components and long persistence within cells [59, 60]. Transfection via mcDNA can achieve efficiencies greater than 50% [48, 60, 105], surpassing lentivirus methods (44% vs. 74% CAR+ cells) [106]. The potential of this technology is bolstered by research demonstrating that CD19.CAR‐T cells produced with mcDNA have remarkable and sustained effects compared to viral‐based T cells. Nonetheless, removing bacterial components from plasmids can be complex and often results in low yields and contamination with bacterial genomic DNA (Figure 3c) [95].

All previously mentioned approaches have been utilized to engineer T cells ex vivo. NPs offer an extremely valid and efficient technique for the specific transfection in vivo of T cells. In 2017, Smith et al. published an interesting article demonstrating the efficiency of polymeric NPs loaded with CAR‐encoding plasmids to induce the expression of CAR on T lymphocytes in vivo, with specificity of nanostructures mediated by an anti‐CD3 antibody [107]. Since then, numerous other platforms have been proposed, including lipid NPs [108, 109], nanotubes [110], and various polymeric nanostructures [111, 112]. However, NPs are not without limitations; scaling‐up manufacturing processes can be complex, and the potential toxicity of NPs must be carefully considered (Figure 3d) [113]. Finally, non‐viral methods for producing CAR‐T cells can be efficiently combined. For instance, mcDNA encoding SB transposase and transposons can achieve transfection efficiencies of up to 65% [114]. One promising approach is represented by NPs that can be efficiently loaded with transposons [107, 115], CRISPR/Cas9, or mcDNA vectors.

## 5 Cytotoxicity of CAR‐T Cells

The initial step in evaluating the killing ability of CAR‐T cells in vitro is conducting a cytotoxicity assay. To date, four main assays have been described in the literature: the chromium (^51^Cr) release assay, flow cytometry (through live/dead staining), luciferase activity, and cell detachment (impedance). Each of these methods has its own advantages and disadvantages [116].

The ratio of effector to target (E:T) cells is the most crucial parameter influencing the efficacy of CAR‐T cells in vitro. Various E:T ratios have been tested in the literature, such as 1:1 [51, 58, 88], 3:1 [74], 5:1 [50, 89], 10:1, and 20:1 [59], with studies evaluating the percentage of cancer cells killed in vitro as well as the release of cytokines. Due to the differences in antigens, the nature of solid versus liquid tumors, and the specifics of CAR generation, there is no consensus on optimal conditions for each CAR‐T and cancer cell combination. Reported E:T ratios have led to the killing of 40 to 95% of both liquid and solid tumor cells. The literature well documents dose‐dependent lysis of tumor cells; however, this finding is primarily applicable to in vitro experiments. In clinical settings, the dosage of CAR‐T cells is rarely determined based on in vitro or preclinical testing (such as murine models). Instead, it is usually adjusted according to body weight, without regard to the number of tumor cells present. This is partly due to the high E:T ratios required in short‐term in vitro assays (lasting 24–48 h) to achieve effective cell lysis. In some cases, an inverse correlation has been observed between the in vitro and in vivo anti‐tumor functions of CAR‐T cells. For instance, variant III epidermal growth factor receptor (EGFR‐vIII) CAR‐T cells have shown functionality at an E:T ratio of 3:1 in vitro, whereas in vivo, the most effective E:T ratio is 1:22.5 [74]. This inconsistency can be attributed to the difficulty in distinguishing CAR‐T cells that are prone to exhaustion.

Additionally, during the in vivo process of tumor eradication, T cells undergo multiple rounds of killing in response to a significant tumor burden. Recent studies have highlighted the potential of in vitro assays to evaluate the repetitive tumor‐killing capacity of CAR‐T cells by rechallenging the co‐culture with fresh tumor cells [117, 118].

### 5.1 Cytokine Production

After interacting with cancer cells, T cells release inflammatory cytokines, granzyme, and perforin to exert anti‐tumor effects. Therefore, in addition to cytotoxicity, cytokine quantification is one of the most important metrics for determining the in vitro function of CAR‐T cells. The enzyme‐linked immunosorbent assay (ELISA) is a widely used method for measuring cytokine release from CAR‐T cells. The most commonly quantified cytokines include interferon‐gamma (INF‐γ) [37, 41, 46, 49, 50, 59, 89], IL‐2 [37, 41, 46, 49, 50, 59], and tumor necrosis factor‐alpha (TNF‐α) [37, 41, 46, 49, 50, 89], along with granulocyte‐macrophage colony‐stimulating factor (GM‐CSF) [46, 62, 84, 89] and Granzyme B (GZMB) [62, 88, 89]. Other cytokines such as IL‐4 [41, 62], IL‐10 [37, 41], IL‐17 [88], macrophage inflammatory protein (MIP‐1), TNF‐related apoptosis‐inducing ligand (TRAIL), and sFas ligand [89] have also been studied.

An alternative to ELISA is the enzyme‐linked immunospot assay (ELISpot test), which is known for its high reproducibility, accuracy, and sensitivity—20–200 times more than traditional ELISA [119]. Historically, ELISpot has been successfully used to detect INF‐γ [120]. Recently, a novel multicolor fluorescent spot assay (MFSA) was developed to detect IL‐10, IL‐2, IL‐4, IL‐5, GZMB, and TNF‐α at the single‐cell level [121].

Flow cytometry and intracellular staining (ICS) are sensitive and specific methods that enable the determination of cytokine production at the single‐cell level. This approach is one of the most frequently used methods for evaluating T cell responsiveness. Flow cytometers can analyze multiple fluorophores simultaneously, allowing several parameters to be assessed concurrently. This method is also considered the fastest, simplest, and most cost‐effective way to analyze cytokine production [122]. The literature indicates that various cytokines have been tested in CAR‐T cells using flow cytometry and ICS analyses [37, 41].

## 6 Automation of CAR‐T Cell Production

The success of CAR‐T cell therapies has heightened the demand for a large‐scale production method that is robust, sterile, reproducible, standardized, and cost‐effective. In addition to biological modifications, the development of advanced manufacturing biotechnologies is essential to ensure the quality and standardization of products, given the increasing requirements for these products. Currently, semi‐automated cell processing systems that comply with good manufacturing practices (GMP) are available for the production and application of CAR‐T therapies [36, 56, 123]. One strategy involves connecting various devices that perform different automated sub‐steps in the CAR‐T cell production process [124]. While there are various approaches, two main platforms are commonly used: CliniMACS Prodigy (Miltenyi Biotec, Germany) and Cocoon (Lonza, Basel, Switzerland). The CliniMACS Prodigy provides a closed automated system capable of producing CAR‐T cells in an unclassified area outside of a cleanroom while still adhering to GMP standards [38]. On the other hand, Cocoon can produce autologous CAR‐T cell therapies in both controlled and uncontrolled environments, ensuring a sterile growth environment. This platform is promising for the automated production of clinical‐scale cell treatments, yielding CAR T‐cell products that are either superior to or comparable with those produced using manual methods. The adoption of this technology also results in reduced production costs for therapeutics [125].

## 7 Conclusions

The effectiveness of CAR‐T cells lies in their strong cytotoxicity against tumor cells, a characteristic that has revolutionized the treatment of hematological diseases. Despite the promising results that have led to substantial investments from companies in this field, academic institutions remain crucial for discovering new targets and producing autologous CAR‐T cells [21]. Developing efficient CAR‐T cells relies on three key factors: selecting a specific and efficient target, optimizing the manufacturing process, and managing toxicity. Tumor‐specific antigens (TSAs) are highly immunogenic and offer new promising targets for personalized tumor immunotherapy. However, identifying TSAs can be complex. Fortunately, recent advances are accelerating the development of therapies to target these antigens [126]. Cancer patients often express tumor‐associated antigens (TAAs), but the high specificity of CAR‐T cells for these antigens can lead to unintended targeting of healthy tissues where these antigens are also present, leading to toxicity. Toxicity is a significant limitation of CAR‐T cell therapy, leading to the development of various engineering strategies to mitigate these adverse effects. One approach involves the induction of suicide genes or “off switches” which allow for the irreversible deactivation of CAR‐T activity as soon as excessive cytokine release is detected. Other strategies include the use of an apoptosis‐triggering fusion protein‐iCas9 (inducible Cas9), which facilitates the rapid and permanent depletion of CAR‐T cells to prevent uncontrolled cytokine release. Additionally, small molecule‐assisted shutoff CARs (SMASH‐CARs) and switch‐off CARs (SWIFF‐CARs) focus on regulating the expression of CAR on the surface of T cells [127]. Furthermore, one study suggested the use of dasatinib, a tyrosine kinase inhibitor, as a quick and reversible method to inhibit CAR‐T cell activation [128].

**FIGURE 4:**
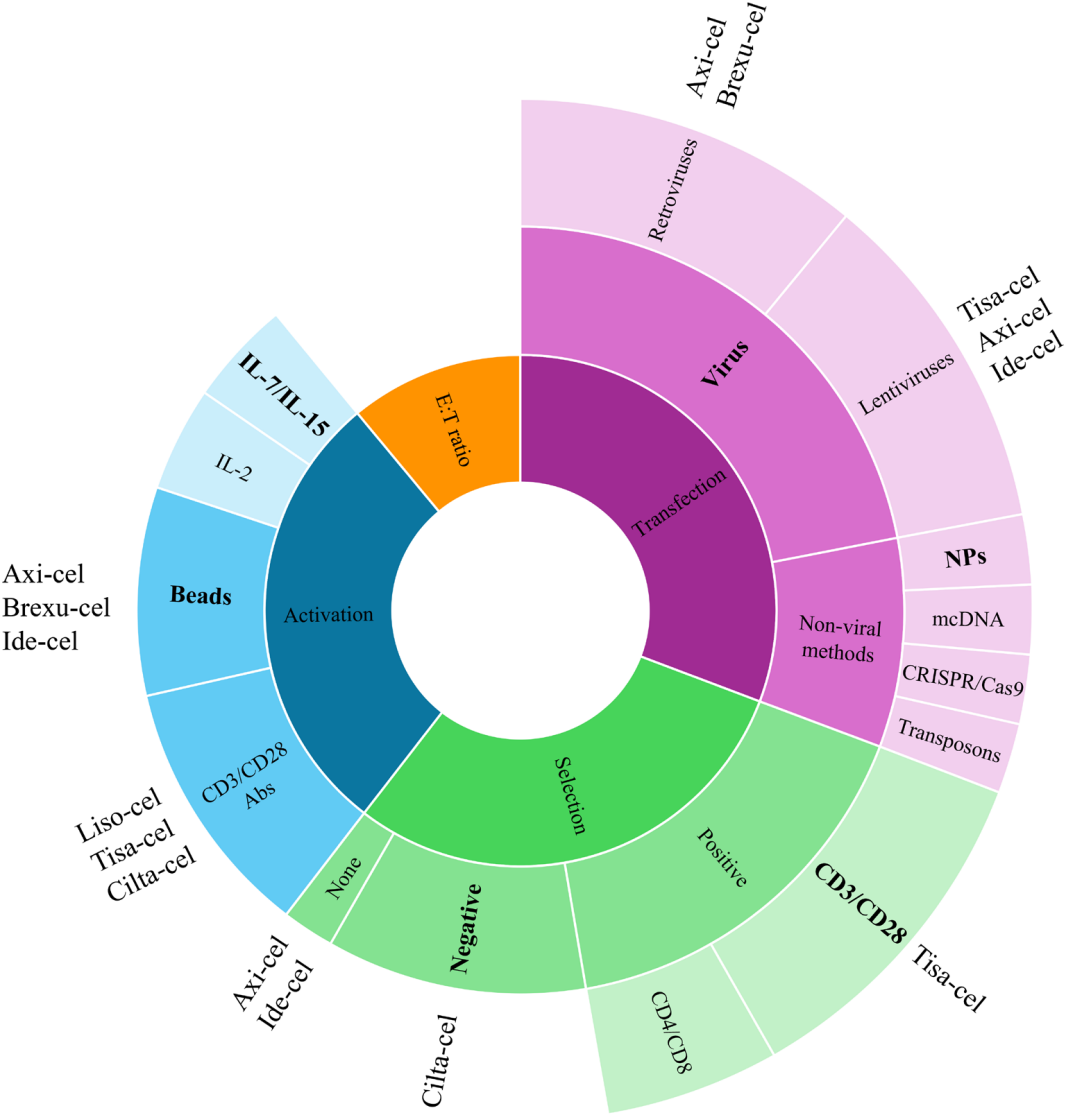
Schematic representation of the critical steps in CAR‐T cell production. CRISPR/Cas9, clustered regularly interspaced short palindromic repeats; E:T, effector‐target; IL, interleukin; mcDNA, minicircle DNA; NPs, nanoparticles.

Another important aspect is CAR‐T cell manufacturing, which is the focus of this review. Although a large amount of information has been published on this technology, much of it remains unclear due to the lack of standardized protocols. The first critical step in CAR‐T cell production is the starting material: purified T lymphocytes versus PBMCs (Figure 4).

While the efficacy of transfecting PBMCs has been proven, the enrichment of T lymphocytes is generally preferred. The second crucial step is the activation of T cells, which is essential for increasing the number of T lymphocytes and preparing them for viral infection, particularly for γ‐retroviruses. Various methods have been used to activate T lymphocytes, including anti‐CD3/CD28 Abs and beads conjugated to these Abs, with the bead method proving to be the predominant choice in preclinical trials. Clinically, both methods are used. The third step is the differentiation state of CAR‐T cells, as it significantly influences their efficacy. IL‐2 has been widely used in both preclinical and clinical studies due to its potent mitogenic activity and ability to promote effector T‐cell proliferation [129]. However, IL‐2‐stimulated T cells often exhibit low efficacy in vivo. Therefore, a memory stem cell phenotype is preferable and can be achieved by stimulating T cells with IL‐7 and IL‐15 [71]. Nonetheless, the literature lacks consensus on the percentage of memory stem cells obtained after stimulation. The fourth crucial step is the transduction of activated lymphocytes. Various viral and non‐viral methods (including transposons, CRISPR/Cas9, mcDNA, and the latest nanodevice‐based approaches) have been developed to minimize the risk of oncogenic insertions associated with viral infections and to improve gene transfer in non‐viral methods. Viral techniques remain the predominant choice for gene transfer into cells. After the production of CAR‐T cells, their efficacy and toxicity must be evaluated in vitro by quantifying single or multiple cytokines through flow cytometry, ELISA, and/or ELISpot.

In vivo experiments are crucial for evaluating the effectiveness of the CAR‐T cell products, as results obtained in vitro only partially reflect the complexity of the tumor in vivo and can sometimes yield inconsistent results [74].

## Author Contributions

M.D.B. and S.C. conceived the original idea; S.C. and M.R. wrote the manuscript; and G.T. and M.D.B. revised the article. All authors have read and approved the final manuscript.

## Ethics Statement

The authors have nothing to report.

## Conflicts of Interest

The authors declare no conflicts of interest.

## Data Availability

Data sharing not applicable to this article as no datasets were generated or analysed during the current study.
